# Severity Prediction over Parkinson's Disease Prediction by Using the Deep Brooke Inception Net Classifier

**DOI:** 10.1155/2022/7223197

**Published:** 2022-05-31

**Authors:** R. Sarankumar, D. Vinod, K. Anitha, Gunaselvi Manohar, Karunanithi Senthamilselvi Vijayanand, Bhaskar Pant, Venkatesa Prabhu Sundramurthy

**Affiliations:** ^1^Department of Electronics and Communication Engineering, QIS Institute of Technology, Ongole 523 272, Andhra Pradesh, India; ^2^Department of Computer Science and Engineering, Saveetha School of Engineering, Saveetha Institute of Medical and Technical Sciences, Chennai, India; ^3^Department of Electronics and Instrumentation Engineering, Easwari Engineering College (An Autonomous Institution), Chennai, India; ^4^Ja Secure Pte Ltd, Singapore; ^5^Department of Computer Science and Engineering, Graphic Era Deemed to Be University, Bell Road, Clement Town, Dehradun 248002, Uttarakhand, India; ^6^Department of Chemical Engineering, Addis Ababa Science and Technology University, Addis Ababa, Ethiopia

## Abstract

Parkinson's disease (PD) is a neurodegenerative illness that progresses and is long-lasting. It becomes more difficult to talk, write, walk, and do other basic functions when the brain's dopamine-generating neurons are injured or killed. There is a gradual rise in the intensity of these symptoms over time. Using Parkinson's Telemonitoring Voice Data Set from UCI and deep neural networks, we provide a strategy for predicting the severity of Parkinson's disease in this research. An unprocessed speech recording contains a slew of unintelligible data that makes correct diagnosis difficult. Therefore, the raw signal data must be preprocessed using the signal error drop standardization while the features can be grouped by using the wavelet cleft fuzzy algorithm. Then the abnormal features can be selected by using the firming bacteria foraging algorithm for feature size decomposition process. Then classification was made using the deep brooke inception net classifier. The performances of the classifier are compared where the simulation results show that the proposed strategy accuracy in detecting severity of the Parkinson's disease is better than other conventional methods. The proposed DBIN model achieved better accuracy compared to other existing techniques. It is also found that the classification based on extracted voice abnormality data achieves better accuracy (99.8%) over PD prediction; hence it can be concluded as a better metric for severity prediction.

## 1. Introduction

Neurological condition Parkinson's disease (PD) is based on dopamine receptors. The most common symptom of Parkinson's disease is difficulty in moving. As a result, a person's gait may become glacial. There are both motor (movement) and nonmotor symptoms associated with Parkinson's disease, which progresses over time. In addition to the numerous similar symptoms, each person will have their own unique experience and manifestation of the disease [1]. When a person has Parkinson's disease, they seem inflexible and stiff-legged. In certain cases, a Parkinson's patient may seem to “freeze up” for a brief period of time [2]. The substantia nigra, which contains dopamine-containing cells, dies in Parkinson's disease, a progressive neurodegenerative disorder. Parkinson disease cannot be reliably distinguished from other disorders with comparable clinical presentations by any test that is consistently trustworthy [3]. Based on the patient's medical history and examination, the diagnosis is essentially a clinical one. Individuals with Parkinson's disease often exhibit the basic symptoms and signs associated with Parkinsonism, notably lack of movement, slowness of movement, stiffness (wrist, shoulder, and neck), and rest tremor (a tremor that occurs when the patient is resting) (imbalance of neurotransmitters, dopamine and acetylcholine). More uncommon disorders, such as numerous cerebral infarctions and progressive supra nuclear palsy (PSP), may also lead to Parkinsonism, as can the usage of certain medications (MSA) [4]. Parkinson's disease is primarily a movement condition, although additional deficits, such as depression and dementia, are common. Autonomic disturbances and pain may develop later, and the disease worsens to the point where the individual becomes severely disabled, resulting in a diminished quality of life [5]. Indirectly, relatives and caregivers may be impacted. Shaking, trouble moving, behavioural issues, dementia, and sadness are all signs of this condition. “Parkinsonism” or a “Parkinsonian Syndrome” is the collective term for the primary motor symptoms [6]. It is possible to identify changes in patients' voices by analysing their voice data. As the condition worsens, the patient's voice starts to stammer and eventually breaks down completely. Unstructured data, such as voice and audio signals, may be efficiently analysed using deep learning techniques [7]. Classification and feature selection in deep neural networks are made possible by the employment of numerous layers of neurons layered on top of one another. “Severe” and “not severe” speech data are analysed using a deep learning-based deep brooke inception net in this research.

The paper's arrangement is as follows: Related work is summarized briefly in Section 2. Problem statement is described in Section 3. The implemented methods for cytokine production prediction are presented in Section 4. The experimental results are introduced in Section 5.  Section 6 concludes the paper.

## 2. Related Works

Predicting Parkinson's disease in a patient is well-studied, but the severity of the illness is less well-studied. Various machine learning and deep learning approaches have been used in these projects, some of which are explained in the following sections [8], in which smartphone sensors were used to provide an objective severity score for Parkinson disease, using machine learning. Dopaminergic therapeutic response was determined using this score, which captured day-to-day symptom changes and was well associated with existing standard rating scales. The gait cycle, which can be broken down into several stages and periods to assess normative and aberrant gait [9], has been presented as a unique way to diagnose PD utilising the gait analysis. It was first necessary to reduce noise caused by variations in the subject's body's alignment during measurement by employing a Chebyshev type II high pass filter with a cutoff frequency of 0.8 Hz. Using peak detection and pulse duration measurement methods, the filtered data was utilised to extract several gait parameters. Gait detection algorithms were adjusted to the specific needs of each user. The heel and toe forces, as well as their normalized values, were derived via the peak detection technique. It was created to extract several temporal parameters, such as the stance and swing phases and the stride time, from the pulse duration. In Parkinson's disease, tremor is a frequent symptom. Body parts move involuntarily with tremors. It is possible for the tremor to begin in a single body part, such as an arm or leg, before spreading to the whole body. In noninvasive prediction systems for Parkinson's disease diagnosis using machine learning and deep neural networks was suggested by the author. People with Parkinson's disease and healthy individuals were classified using machine learning prediction models such as support vector machine, logistic regression, and deep neural network. 70% of the data were used for training, while 30% were used for testing. Model performance was also assessed using criteria such as classification accuracy, sensitivity, specificity, and Matthews' correlation coefficient. The suggested system was tested using data from the UCI machine learning repository, which included 23 characteristics and 195 cases related to Parkinson's disease. In [10] authors have presented a technique for predicting the severity of Parkinson's disease based on Parkinson's Telemonitoring Voice Data Set of patients at UCI. In order to build the neural network, they employed the Python “TensorFlow” deep learning module, suggesting a new method of detecting Parkinson's disease by analysing gait data using deep learning algorithms. Deep Neural Network (DNN) classifier was constructed with the help of a 1D convolutional neural network (1D-Convnet). A real-time speech signal analysis method for Parkinson's disease (PD) diagnosis and severity evaluation was the goal of the author. It should also be possible to include any machine learning or deep learning method into the system's underlying framework. In order to forecast the course of Parkinson's disease, the author developed and validated a radiomics model based on whole-brain white matter and clinical characteristics. In [11] researchers used activities with low (reading) and high (retelling) processing demands to try to uncover which speech features better identify individuals with PD in cognitively diverse, cognitively maintained, and cognitively impaired groups, to assess prosodic, articulatory, and phonemic identification aspects using support vector machines. In PD patients gait signals were evaluated to determine the severity of their condition. Recurrence plots were created using gait signals and characteristics were retrieved. Several categorization studies were carried out using machine learning methods. The severity of Parkinson's disease was predicted using binary and multiclass classifications depending on gender. In [12] for the categorization of Parkinson's disease, the author presented a unique technique based on data partitioning and the principal component analysis (PCA) algorithm for feature selection. EnKNN is shown to be an ensemble K-nearest neighbour. Base classifiers are constructed using the K-nearest neighbour approach, and the weight of each base classifier is generated using the G-mean score and the F-measure of each extracted feature. In [13] an improved tremor severity categorization method is presented. In this case, signal processing and resampling methods such as oversampling, undersampling, and a hybrid combination are used. Artificial neural network based on multilayer perceptrons (ANN-MLP) and random forest classifiers use resampling approaches (RF). In the classification, six different machine learning (ML) methods are applied. Different classification methods exist, including Stochastic Gradient Descent (SGD) classifiers, Extreme Gradient Boosting (XGB), logistic regression, random forest, K-nearest neighbour (KNN) classifiers, and decision tree (DT) classifiers. The goal of their study is to decrease the dataset's complexity by classifying Parkinson's disease using human speech signals and extracting key characteristics. Human vocal signals are then examined to determine the PD patient's voice strength and frequency. In [14] the author utilised voice measures from 31 participants, 23 of whom had been diagnosed with Parkinson's disease (PD), using a data set obtained from Kaggle. Each person's 195 voice recordings were included in the data collection, which comprised 22 distinct characteristics related to voice measures. In the preprocessing of the data, they deleted the associated characteristics and utilised 10 noncorrelating attributes (0.7) together with the individual status (0 and 1 for healthy and PD, respectively). This data was preprocessed in such a way that the number of normal vs. PD subjects was determined to be identical throughout the training and testing data sets. Random forest, XGBoost, SVM, and decision tree (DT) models were used to analyse the data set for four supervised classification machine learning (ML) models. In [15] automating the diagnosis of Parkinson's disease using sEMG signals was the primary goal of the author, who used pretrained deep transfer learning structures and standard machine learning models. For the most part, they built the discriminative feature vectors by stacking the extracted features from three deep pretrained architectures: AlexNet, VGG-f, and CaffeNet. Overfitting was avoided, and the resilience to additional noise of various degrees was increased, thanks in part to the correct features stacked from all three deep structures. A new combination of subset feature selection approaches, such as receiver operating characteristic (ROC), entropy, and the signal-to-noise (SNR) processes, was then offered as a way to minimize feature size. Their final tool was an SVM with an RBF kernel to help them diagnose Parkinson's disease.

## 3. Problem Statement

The use of EMG signal analysis for the diagnosis of Parkinson's disease (PD) has not been employed despite several researches reporting acceptable classification accuracy using deep learning. There are certain drawbacks to DL, which makes it challenging to combine with other prediction methods. Massive volumes of data are required, as are sophisticated data models, and there is little guidance from normal theory in selecting an acceptable deep learning algorithm. Additionally, any event prediction technique must be accurate in order to analyse EMG data. Accuracy can only be ensured with the right and effective features. To sum up, there are a total of two key obstacles to overcome:

1. There is a need to better understand the nature of PD condition that might cause the illness based on speech signals that are received.
2. Models created to aid in diagnosis of Parkinson's disease must be able to categorize the ailment.

## 4. Proposed Work

**Figure 1:**
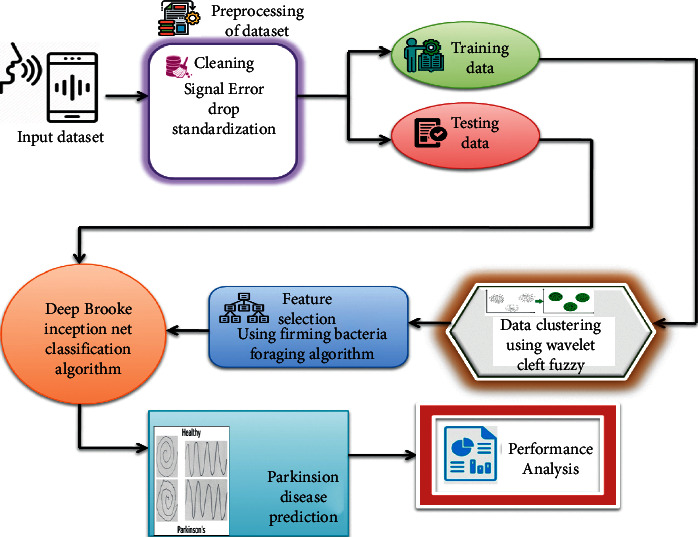
Schematic representation of the suggested methodology.

Deep learning may be used to forecast how severe Parkinson's disease is, as seen in Figure 1. Voice data from individuals with Parkinson's disease is first gathered for analysis. Signal error drop standardization is then used to normalize the data. An input layer, hidden layers, and output layer are created in the following phase of deep neural network architecture. A predetermined amount of input data characteristics determines how many neurons are in the input layer. Neurons in the output layer correlate to “severe” and “nonsevere” categories. The normalized data is sent into a deep neural network for training and testing. UCI Machine Learning Repository's Parkinson's Telemonitoring Voice Data Set was utilised. 42 patients' biological voice measures are included in the collection. Subject number, age, gender, time interval, Motor UPDRS, Total UPDRS, and 16 biomedical voice measurements are some of the data's properties. These patients' voices may be heard in 5,875 audio files in the collection. CSV files are used to store the information. Each patient provides an average of around 200 recordings (identification can be done through the first attribute-subject number).

### 4.1. Preprocessing

The linear transformation of the initial random range of data is provided by the signal error drop approach. The recommended normalization is a straightforward method with a clearly stated error threshold. According to the standardization method, the data should be arranged in this way.

(1) $$\begin{array}{c} P^{,}=\left(\frac{P-\min\text{ value of }P}{\max\text{ value of }P-\min\text{ value of }P}\right)*\left(d-C\right)+C, \end{array}$$

where *P*′ contains input data, *d* is the cross layer error, and *C* is the inbound matrix.

Here the unstructured data can be standardized using *z*-score parameter, as per given formula.

(2) $$\begin{array}{c} S_{i^{\prime }}=\frac{q_{i}-\bar{S}}{\text{std}\left(S\right)}, \end{array}$$

*s*_*i*_′ is the structured data. *q*_*i*_ is value of the standard variable, where

(3) $$\begin{array}{c} \text{std }\left(S\right)=\sqrt{\frac{1}{\left(m-1\right)}\sum_{i=1}^{m}{\left(q_{i}-\bar{S}\right)}^{2}},\\ \bar{S}=\frac{1}{m}\sum_{i=1}^{m}q_{i}. \end{array}$$

The error drop of a finite *N* longitudinal series is described as

(4) $$\begin{array}{c} y\left(s\right)=\sum_{D=0}^{D-1}Y\left[D\right]Q_{D}^{kn},\;K=0,1,2\ldots ,D-1, \end{array}$$

where *Q*_*D*_^*kn*^ is representing a complex value

(5) $$\begin{array}{c} Q_{D}^{kn}=e^{iI2\pi K/N}=\text{ Cos}\left(\frac{2\pi k}{N}\right)-i\text{Sin}\left(\frac{2\pi k}{N}\right). \end{array}$$

Nearly identical is the reverse transform, which returns the common matrix from the frequency domain. The frequency spectrum of an arbitrary signal *y*(*D*) inside [0; *q*] is shown below. To show that a signal's PD is zero-order, Fourier's zero-order functions are used:

(6) $$\begin{array}{c} Y\left(PD\right)=\sum_{j=1}^{n}c_{ji_{0}}\left(\frac{\beta_{j}n}{N}\right),\;n=0,1,\ldots ,N-1. \end{array}$$

Here *c*_*j*_ can be represented as the error coefficients of *y*(*s*) that can be mathematically represented as

(7) $$\begin{array}{c} c_{j}=\frac{2}{n^{2}}\left(j_{2{\left(\beta_{j}\right)}^{2}\sum_{D=2}^{n}nx\left(n\right)}\right.j_{1}\left(\frac{\beta_{j}n}{N}\right), \end{array}$$

where *j*_1_and*j*_2_ represent the first- and the second-order equations of the error functions. The positive roots of the matrix functions can be represented by *β*_*j*_ with *j* = 1, 2,…, *N*. The order of the *j* th function FFS coefficients is represented as

(8) $$\begin{array}{c} \beta_{j}=2\pi f^{j}\left[\frac{D}{D^{\wedge }s}\right]. \end{array}$$

And

(9) $$\begin{array}{c} \beta_{j}\approx \beta -1+\pi \approx j\pi \text{ and.} \end{array}$$

A final value ranging from 0 to 1 may be obtained by standardizing the *Z*-score. Decimal Scaling is the method used to produce a range of −1 to 1. Consequently, with the decimal scaling method,

(10) $$\begin{array}{c} \beta^{i}=\;\frac{q}{10^{j\;}}. \end{array}$$

The maximum standardized score obtained was illustrated as follows:

(11) $$\begin{array}{c} \max\;ME_{ROI}=\;\begin{array}{c} \max\\ \beta i \end{array}\left(\frac{\sum_{i\in ROI}\left|m_{i}-\tilde{m_{i}}\right|}{N_{ROI}}\right). \end{array}$$

### 4.2. Data Clustering

It is the theory that related things may be grouped together into clusters, where similarities inside the cluster are considerable and differences between clusters are minimal. WCF clustering is built on this concept. A feature attribute data matrix is a data package for input. Parts that share characteristics may be categorized as components, and the properties of those components can be referred to as elements. Despite the fact that WCF needs less technical examination, WCF tends to be focused on a certain geographic region. The lower degree of technical examination and the concentration on a single region of WCF make it a good fit for many applications. The following equation is used by the WCF method to include all of the data:

(12) $$\begin{array}{c} M_{s,\theta =0\left(p,q\right)}=\sum_{n=1}^{N}\sum_{m=1}^{K}\left\{\begin{array}{cc} 1 & c\\ 0 & \text{otherwise,} \end{array}\right. \end{array}$$

where *M* is the number of iterations, *p*, *q* is the regulatory factor (12). You may determine how far apart two points are using this method. It is possible to perform the grouping based on that.

(13) $$\begin{array}{c} \Sigma u,v\;\frac{p\left(u,v\right)}{1+\left|u-v\right|}-\sum_{n=1}^{N_{vk}}\sum_{m=1}^{M_{vk}}p_{vk}\left(m,n\right).\;\log\;p_{vk}\left(m,n\right)=0, \end{array}$$

where Σ*u*, *v*  = fitness distance.

After the calculation of the fitness distance there is a need to compute the closest centroid,

(14) $$\begin{array}{c} E\left(d,y\right)=\left\{\begin{array}{cc} 1, & \left(d,y\right)\epsilon Q_{g}\\ 0, & \text{ otherwise,} \end{array}\right. \end{array}$$

where *Q*_*g*_={(*d*, *y*) : *g*(*d*, *y*) > *b*}, *b* represents the threshold value, and *E*(*d*, *y*) denotes the centroid map, where

(15) $$\begin{array}{c} E\left(d\right)=\frac{R_{s}\cap T_{s}}{R_{s}\cup T_{s}},\;\;E\left(y\right)=\left|\frac{R_{s}-T_{s}}{R_{s}}\right|. \end{array}$$

Hence *E*(*d*)  = Grouped centroid 1, and *E*(*y*)  = Grouped centroid 2. *R* is the error variance; *f* is the functional nodal characteristics.

After all objects have been assigned, a new calculation of the centroids' positions should be made. Go return to the previous stages and restart the procedure as soon as the centroids have stopped moving; there should be different groups if the threshold is lower than the similarity.

### 4.3. Feature Selection

The data are grouped in the form of cluster in the previous step. Here from clustered features the specialized features regarding disease can be isolated using the firming bacteria foraging optimization (FBFO).

(I) It may take many generations to discover the optimal solution if the step size is really small. A global optimum may not be reached with fewer iterations.
(II) To obtain the ideal value rapidly, the bacteria will need a big step size, but its accuracy will be limited. Aside from the chemotaxis stage, the reproduction technique speeds up convergence, while removal and dispersion prevent premature convergence in the FBFO. Instead of removing and disseminating, FBFO uses mutation to achieve variable step size, increased speed, and prevention of premature convergence.
  (16) $$\begin{array}{c} \left(h+1,k\right)=\theta^{h}\left(h+1,k\right)+*r_{1}*C_{1}\left(\theta^{h}\right.\left(h+1,k\right)-\theta_{\text{global}}. \end{array}$$

*θ* ^*h*^(*h*+1, *k*) = Position vector of *i*-th bacteria in *h*-th chemotaxis step and *k*-th reproduction stages.

*θ* ^*i*^ global = Good location in the full search area.

The operation of the mean square chemotaxis that firming the bacterial foraging method in order to reduce the steps in the feature selection approach is as follows:

(17) $$\begin{array}{c} \text{chemo}=\frac{1}{mn}\sum_{j=1}^{mn}{\left(z\left(x,y\right)-p\left(x,y\right)\right)}^{2}. \end{array}$$

The size of the clustered data is shown by the MN in this case.

The fitness distance between two groups is then calculated using a set of five potential lengths. In order to calculate *D*0 and *D*1, the centroid of two clusters is used.

(18) $$\begin{array}{c} D0={\left({\left(\overset{⟶}{a_{0_{1}}}-\overset{⟶}{a_{0_{1}}}\right)}^{2}\right)}^{1/2},\\ D1=\left|\overset{⟶}{a_{0_{1}}}-\overset{⟶}{a_{0_{1}}}\right|=\sum_{i=1}^{d}\left|{\overset{⟶}{a_{0_{1}}}}^{\left(i\right)}-{\overset{⟶}{a_{0_{1}}}}^{\left(i\right)}\right|, \end{array}$$

Encircling or spiraling bacteria is a random number that shows how likely it is that the bacteria will be relocated; *D* ∈ [0, 1]. An ant may be able to climb the food chain by utilising an unintentional search agent instead of the optimal one.

(19) $$\begin{array}{c} F=\left|D⊙\;Y_{rand}\;-\;Y\left(IF\right)\right|, \end{array}$$

where *Y*_rand_ represents a random location vector selected from the existing population. Hence here as per bacteria food searching procedure the best fitness abnormal features can be clumped up that can be given as a input for the classification process.

### 4.4. Classification

The deep brooke inception net is used in this model. Using this method, there is no reduction in the weight of the network. For the model's development, we employed convolutional layers (CL), global average pooling, fully connected layers (FC), and classification layers (CL + FC). Using the experimental data, a network model is trained, and that model is then put to use in the classification process. There are two major downsides to increasing network depth in deep learning: gradient disappearance and gradient explosion. When dealing with the gradient problem, data initialization and regularisation is a frequent method, but it has the unintended consequence of lowering network performance. The complexity has climbed, but so has the quantity of mistakes. By adding a residual element to the network, network performance will be enhanced and the gradient problem will be solved. Model layers may be degraded to minimize performance degradation by mapping the layers underneath the deep network in a similar fashion. The identity mapping function is all that is required to perform the classification operation in the network model,

(20) $$\begin{array}{c} G\left(x\right)=f\left(x\right)+x, \end{array}$$

where *G*(*x*) represents the same map or mapping to summing, *f*(*x*) represents the mapping of network before sum, and *x* symbolizes the input to prelayer of model. As long as *f*(*x*) is equal to zero, *G*(*x*) is equal to *x* as well. In order to discover the residual function, this network must be transformed in accordance with the following equation:

(21) $$\begin{array}{c} f\left(x\right)=G\left(x\right)-x. \end{array}$$

Considering the forward process, the end result shows a straightforward path from *P* layer to *P* layer.

(22) $$\begin{array}{c} G_{s+1}=d_{s}+F\left(G_{s},W_{s}\right),\\ d_{s+2}=d_{s+1}+F=G_{s}+F\left(G_{s},W_{s}\right)+F\left(d_{s+1,\;}W_{s+1}\right). \end{array}$$

Here,

(23) $$\begin{array}{c} G_{s}=G_{s}+\sum_{q=s}^{P-s}F\left(G_{q},W_{q}\right), \end{array}$$

where *W*_*q*_ represents the equivalent mapping method. There must be a factor for each residual element to estimate how big of an influence a future input will have. For residual elements, the forward process is linear. In the first major characteristic of the residual network, the gradient disappearing problem is solved by the reverse updating. This means that a gradient from the *P* layer may be transferred reliably to the *p* layer by propagating the residual network backwards. Specifically, the method is outlined in the following equation:

(24) $$\begin{array}{c} \frac{\partial Ε}{\partial_{G_{l}}}=\frac{\partial Ε}{\partial_{G_{L}}}\frac{\partial d_{L}}{\partial_{G_{L}}}=\frac{\partial Ε}{\partial_{G_{l}}}\left(1+\frac{\partial }{\partial G_{l}}\sum_{q=l}^{L-1}F\left(G_{q},W_{q}\right)\right). \end{array}$$

The residual element's structural design protects the network against gradient disappearance during backpropagation training. Additionally, if the overall network performance suffers, the redundant network layer may do a similar mapping, enabling residual learning to be applied in practice. Using CLs, feature maps may be built and detailed features can be extracted. The ReLU function is used as an activation function in these layers, allowing neurons to analyse more features.

(25) $$\begin{array}{c} \text{ReLU}\left(G\right)=\left\{\begin{array}{cc} G, & \text{if }G>0,\\ 0, & \text{if }d\leq 0. \end{array}\right. \end{array}$$

The number of parameters in the model is significantly reduced once the global average pooling layer is included, reducing the chance of overfitting. It also sums up the spatial information, making it more resistant to translations of the input. FC receives a condensed version of the feature map. We must employ a binary classification system to differentiate STB from LC. Thus, the classification layer employs the sigmoid function in this manner.

(26) $$\begin{array}{c} y=F\left(d,\left\{W_{q}\right\}\right)+d, \end{array}$$

where *d* and *y* represent the input and output vectors and *W*_*q*_ represents the equivalent mapping method. The function *F*(*d*, {*W*_*q*_}) denotes the residual function. Hence by using the suggested network model the Parkinson features can be identified precisely (Algorithm 1).

## 5. Performance Analysis

Using a voice record dataset, the deep Brooke inception net method with a decision is utilised to categorize Parkinson disease patients. Assessment testing may take many forms, including medical exams, physical symptoms (such as tremors in the hands and feet, tremors in the voice, and other markers of Parkinson's disease), and even physical signals. An accurate diagnosis is essential in medicine, and screening tests play an important role in doctors' medical treatment opinions. Fortunately, the properties of the diagnostic tests may be measured; this is a blessing. The best possible diagnosis for a certain sickness state may be determined based on these factors. It is usual practice to use sensitivity, specificity, and accuracy to describe diagnostic tests. They are critical for determining how accurate and efficient a test is. The categorization process' quality rate must also be determined. Testing involves locating the diseased individual in a speech dataset using the provided approach and evaluating the results using performance metrics including sensitivity, specificity, and accuracy. False positive, true negative, and false negative are some of the terms that must be calculated in order to get at these measurements.

**Figure 2:**
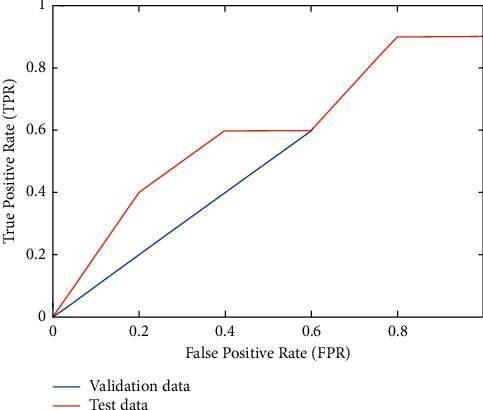
False positive vs. true positive rate.

Positive outcomes are those that the model accurately predicts. Additionally, the model's ability to properly anticipate which outcomes are bad is referred to as a “true positive.” When the model mistakenly predicts a positive class, this is known as a false positive. On the basis of training and validation data, the proposed technique had a greater true predictive value than the alternative, as shown in Figure 2. To prove the efficiency of the suggested methodology it can be compared with the conventional methods [15].

### 5.1. Accuracy

Accuracy is defined as the percentage of true positive or true negative results in a population. It measures the precision with which a test result identifies a certain disease state.

(27) $$\begin{array}{c} \text{Accuracy}=\frac{\left(\text{TP}+\text{TN}\right)}{\left(\text{TP}+\text{FN}+\text{TN}+\text{FP}\right)}. \end{array}$$

**Figure 3:**
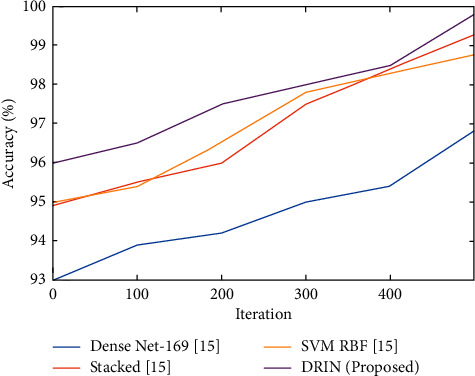
Comparison of accuracy (%) for existing and proposed method.

Existing and proposed methods for assessing their correctness are shown in Figure 3. It is obvious from the graph that the created method is more accurate than the current methods in predicting Parkinson disease.

### 5.2. Sensitivity

**Figure 4:**
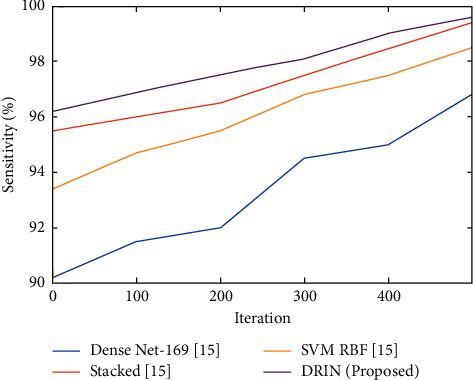
Comparison of sensitivity (%) for existing and proposed method.

The probability of the test identifying an abnormal case using a suggested classifier was illustrated in Figure 4. Sensitivity is the percentage of true positives correctly recognized by a diagnostic test. It shows how successfully the screening detects a problem.

(28) $$\begin{array}{c} \text{Sensitivity}=\frac{\text{TP}}{\left(\text{TP}+\text{FN}\right)}. \end{array}$$

Figure 4 shows the comparative evaluation of sensitivity for the current and the suggested approaches. The suggested technique acquires 99.7% sensitivity which was very high when compared to other existing methods.

### 5.3. Specificity

The probability of finding the normal test case among all other cases in normal is called specificity. Specificity is the proportion of the true negatives correctly identified by a diagnostic test. It suggests how good the test is identifying normal (negative) condition.

(29) $$\begin{array}{c} \text{Specificity}=\frac{\text{TN}}{\left(\text{TN}+\text{FP}\right)}\text{.} \end{array}$$

**Figure 5:**
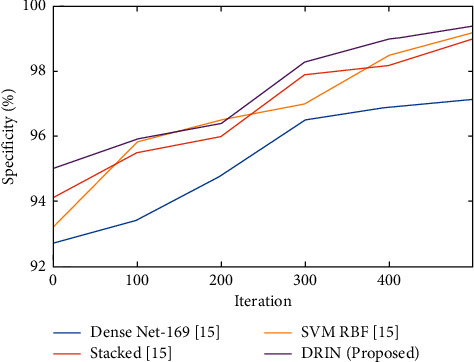
Comparison of specificity (%) for existing and proposed method.

The comparison of specificity for the conventional and recommended procedures is shown in Figure 5. When compared to current approaches, the graph shows that the recommended method performs better in terms of specificity.

Specificity defines true negative that is many tuples (negative) which are correctly rejected. The suggested methodology acquires 99.5% of specificity rate which was very high when compared to other existing methods.

**Figure 6:**
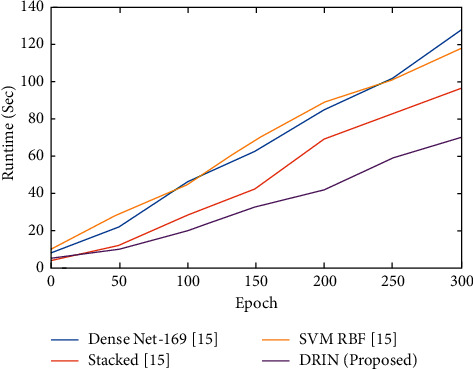
Epoch vs. runtime.

The normalized runtime for each epoch was illustrated in Figure 6. From Figure 6 the suggested prediction process can wind up the process within 65 seconds which was very low when compared to other existing mechanisms. From the overall result obtained it was revealed that the suggested methodology outperforms the other existing mechanisms.

## 6. Conclusion

In this paper, we have implemented a deep neural network to predict the severity of Parkinson's disease. The proposed DBIN model achieved better accuracy compared to other existing techniques. It is also found that the classification based on extracted voice abnormality data achieves better accuracy (99.8%) over PD prediction; hence it can be concluded as a better metric for severity prediction. Although we have used a dataset of high instances, the accuracy of our approach can be further improved by implementing it on a larger dataset, having larger number of instances of each severity class as well as on a combined database of patients' voice data and other patient attributes like gait and handwriting features. This could enhance diagnosis and aid doctors in making prompt intervention decisions in individuals with Parkinson's disease. DBIN preserves a correct capability as a classifier over voice data for various stages of PD, according to the current results.

## Figures and Tables

**Algorithm 1:**
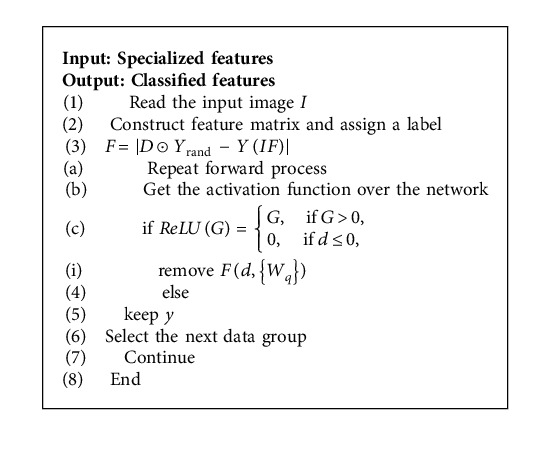
(Deep brooke inception net).

## Data Availability

The datasets used and/or analysed during the current study are available from the corresponding author on reasonable request.
